# The complete mitochondrial genomes for three *Toxocara *species of human and animal health significance

**DOI:** 10.1186/1471-2164-9-224

**Published:** 2008-05-16

**Authors:** Ming-Wei Li, Rui-Qing Lin, Hui-Qun Song, Xiang-Yun Wu, Xing-Quan Zhu

**Affiliations:** 1 Laboratory of Parasitology, College of Veterinary Medicine, South China Agricultural University, Guangzhou, Guangdong Province 510642, ProC; 2 Department of Veterinary Medicine, Agricultural College, Guangdong Ocean University, Zhanjiang, Guangdong Province 524088, ProC; 3 Key Laboratory of Marine Bio-resources Sustainable Utilization, South China Sea Institute of Oceanology, Chinese Academy of Sciences, Guangzhou, Guangdong Province 510301, ProC

## Abstract

**Background:**

Studying mitochondrial (mt) genomics has important implications for various fundamental areas, including mt biochemistry, physiology and molecular biology. In addition, mt genome sequences have provided useful markers for investigating population genetic structures, systematics and phylogenetics of organisms. *Toxocara canis, Toxocara cati *and *Toxocara malaysiensis *cause significant health problems in animals and humans. Although they are of importance in human and animal health, no information on the mt genomes for any of *Toxocara *species is available.

**Results:**

The sizes of the entire mt genome are 14,322 bp for *T. canis*, 14029 bp for *T. cati *and 14266 bp for *T. malaysiensis*, respectively. These circular genomes are amongst the largest reported to date for all secernentean nematodes. Their relatively large sizes relate mainly to an increased length in the AT-rich region. The mt genomes of the three *Toxocara *species all encode 12 proteins, two ribosomal RNAs and 22 transfer RNA genes, but lack the ATP synthetase subunit 8 gene, which is consistent with all other species of Nematode studied to date, with the exception of *Trichinella spiralis*. All genes are transcribed in the same direction and have a nucleotide composition high in A and T, but low in G and C. The contents of A+T of the complete genomes are 68.57% for *T. canis*, 69.95% for *T. cati *and 68.86% for *T. malaysiensis*, among which the A+T for *T. canis *is the lowest among all nematodes studied to date. The AT bias had a significant effect on both the codon usage pattern and amino acid composition of proteins. The mt genome structures for three *Toxocara *species, including genes and non-coding regions, are in the same order as for *Ascaris suum *and *Anisakis simplex*, but differ from *Ancylostoma duodenale*, *Necator americanus *and *Caenorhabditis elegans *only in the location of the AT-rich region, whereas there are substantial differences when compared with *Onchocerca volvulus*,*Dirofiliria immitis *and *Strongyloides stercoralis*. Phylogenetic analyses based on concatenated amino acid sequences of 12 protein-coding genes revealed that the newly described species *T. malaysiensis *was more closely related to *T. cati *than to *T. canis*, consistent with results of a previous study using sequences of nuclear internal transcribed spacers as genetic markers.

**Conclusion:**

The present study determined the complete mt genome sequences for three roundworms of human and animal health significance, which provides mtDNA evidence for the validity of *T. malaysiensis *and also provides a foundation for studying the systematics, population genetics and ecology of these and other nematodes of socio-economic importance.

## Background

Mitochondria are sub-cellular organelles involved in oxidative phosphorylation, offering energy to organisms. They play important roles in cellular metabolism, living and apoptosis. Within these organelles, most metazoan species possess a compact, circular mitochondrial (mt) genome, which varies in size from 14 to 20 kb [1]. The metazoan mt genome usually contains a complement of genes encoding 12–13 protein subunits of the enzymes involved in oxidative phosphorylation, 22 transfer RNAs, and two ribosomal RNAs. There are no introns within genes, and no to limted spacer regions between genes [1,2]. Studying mt genomes has important implications for various fundamental areas, including mt biochemistry, physiology and molecular biology. In addition, mt genome sequences have provided useful markers for investigating population genetic structures, systematics and phylogenetics of organisms due to their maternal inheritance, higher mutation rates than nuclear genes and relatively conserved genome structures [3-5].

Although Nematoda is the second largest animal phylum, to date only over 40 complete mitochondrial DNA sequences of nematode species have been deposited in GenBank™ [6-15]. In the order Ascaridada, the mt genomes of only two species have been sequenced [6,15]. The lack of knowledge of mt genomics for parasitic nematodes in this order is a major limitation for population genetic and phylogenetic studies of the pathogens in the order Ascaridada including the species in the genus *Toxocara*.

*Toxocara canis, Toxocara cati *and *Toxocara malaysiensis *are the common ascaridoid nematodes of dogs and cats, causing significant health problems. Infection of dogs with *T. canis *is quite common, with the prevalence ranging from 5.5% to 64.7% [16-18]. Infection of cats with *T. cati *is also found worldwide, with infection rates of up to 25.2% to 66.2% [19-21]. More importantly, *T. canis *and *T. cati *are of public health significance due to their larvae of being able to invade humans and cause diseases such as ocular larva migrans (OLM), visceral larva migrans (VLM), eosinophilic meningoencephalitis (EME) and/or covert toxocariasis (CT) [22-24]. Although they are of importance in human and animal health, there is still no information on the mt genomes available for any of *Toxocara *species.

The objectives of the present study were to fill some of these knowledge gaps by determining the structure, organization and sequence of the complete mt genomes of *T. canis*, *T. cati *and *T. malaysiensis *of human and animal significance, and to provide mtDNA evidence for the recently described species *T. malaysiensis *[25,26]. Also, the features of mt genomes of the three ascaridoid nematodes, such as the gene arrangements, structures, compositions, as well as translation and initiation codons and codon usage patterns were compared with those of other nematodes in the same order Ascaridata, namely *Ascaris suum *and *Anisakis simplex*. The phylogenetic relationships among these ascaridoid nematodes were also investigated using the protein-coding amino acid sequences.

## Results and Discussion

### General features of the mt genome of three *Toxocara *species

The complete mt genomes of *T. canis*, *T. cati *and *T. malaysiensis *are 14,322 bp, 14,029 bp and 14266 bp in length, respectively. These complete mt genome sequences have been deposited in the GenBank™ under the accession numbers AM411108 (for *T. canis*), AM411622 (for *T. cati*) and AM412316 (for *T. malaysiensis*). All three mt genomes contain 12 protein-coding genes (*cox*1-3, *nad*1-6, *nad*4L, *atp*6 and *cyt*b), 22 transfer RNA genes and two ribosomal RNA genes, but lack an *atp*8 gene (Table 1). These circular genomes are typical of other nematode mitochondrial genomes except for *Trichinella spiralis *in which the *atp*8 gene is encoded. All genes are transcribed in the same direction as found in other members of the secernentean nematodes sequenced to date, but in contrast to *T. spiralis *and *Xiphinema americanus *[8,13].

**Table 1:** Positions and nucleotide sequence lengths of mitochondrial genomes of *T. canis, T. cati *and *T. malaysiensis*, and start and stop codons for protein-coding genes as well as lengths of their predicted amino acid sequences and tRNA gene anticodons (starting from *nad*1)

**Genes**	**Positions and nt sequence lengths (bp)**			**Initiation and termination codons (Ini/Ter)**			**No. of amino acid**			**anticodons**		
	**Tcan**	**Tcat**	**Tmal**	**Tcan**	**Tcat**	**Tmal**	**Tcan**	**Tcat**	**Tmal**	**Tcan**	**Tcat**	**Tmal**

*nad*1	1–873 (873)	1–873 (873)	1–873 (873)	TTG/TAG	TTG/TAA	TTG/TAG	291	291	291			
*atp*6	876–1473 (598)	875–1472 (598)	875–1472 (598)	ATT/T	ATT/T	ATT/T	200	200	200			
trn-Lys	1474–1535 (62)	1473–1535 (63)	1473–1533 (61)							TTT	TTT	TTT
trn-LeuUUR	1536–1590 (55)	1536–1590 (55)	1534–1589 (56)							TAA	TAA	TAA
trn-SerAGN	1591–1642 (52)	1591–1641 (51)	1590–1642 (53)							TCT	TCT	TCT
*nad*2	1643–2486 (844)	1642–2485 (844)	1643–2486 (844)	ATT/T	GTT/T	ATT/T	282	282	282			
tRNA-Ile	2487–2547 (61)	2486–2546 (61)	2487–2549 (63)							GAT	GAT	GAT
tRNA-Arg	2548–2601 (54)	2547–2602 (56)	2550–2604 (55)							ACG	ACG	ACG
tRNA-Gln	2604–2658 (55)	2603–2657 (55)	2605–2659 (55)							TTG	TTG	TTG
tRNA-Phe	2659–2716 (58)	2658–2716 (59)	2662–2718 (57)							GAA	GAA	GAA
*cyt*b	2717–3823 (1107)	2717–3823 (1107)	2719–3825 (1107)	GTG/TAG	ATG/TAA	GTG/TAG	369	369	369			
tRNA-LeuCUN	3824–3879 (56)	3826–3880 (55)	3826–3882 (57)							TAG	TAG	TAG
*cox*3	3880–4647 (768)	3881–4648 (768)	3883–4650 (768)	ATG/TAG	ATG/TAA	GTG/TAG	256	256	256			
tRNA-Thr	4648–4704 (57)	4652–4708 (57)	4651–4705 (55)							TGT	TGT	TGT
*nad*4	4705–5934 (1230)	4709–5938 (1230)	4706–5935 (1230)	ATA/TAG	ATA/TAG	ATA/TAG	410	410	410			
Non-coding region	5935–6045 (111)	5939–6054 (116)	5936–6047 (112)									
*cox*1	6046–7623 (1578)	6055–7632 (1578)	6408–7628 (1581)	TTG/TAG	TTG/TAG	TTG/TAG	526	526	527			
tRNA-Cys	7926–7680 (55)	7635–7688 (54)	7631–7686 (56)							GCA	GCA	GCA
tRNA-Met	7689–7748 (60)	7694–7753 (60)	7694–7753 (60)							CAT	CAT	CAT
tRNA-Asp	7750–7808 (59)	7754–7809 (56)	7755–7813 (59)							GTC	GTC	GTC
tRNA-Gly	7808–7863 (56)	7809–7866 (58)	7813–7869 (57)							TCC	TCC	TCC
*cox*2	7864–8577 (714)	7867–8577 (711)	7870–8580 (711)	GTT/TAG	GTT/TAA	GTA/TAG	238	237	237			
tRNA-His	8576–8631 (56)	8577–8634 (58)	8579–8637 (59)							GTG	GTG	GTG
*rrn*L	8632–9589 (958)	8635–9589 (955)	8638–9592 (955)									
*nad*3	9590–9925 (336)	9590–9925 (336)	9593–9928 (336)	TTG/TAG	TTG/TAG	TTG/TAG	112	112	112			
*nad*5	9927–11508(1582)	9926–11507 (1582)	9931–11512(1582)	ATA/T	ATA/T	ATG/T	528	528	528			
tRNA-Ala	11509–11563 (55)	11508–11565 (58)	11513–11570 (58)							TGC	TGC	TGC
tRNA-Pro	11567–11623 (57)	11566–11621 (56)	11573–11628 (56)							TGG	TGG	TGG
tRNA-Val	11624–11679 (56)	11622–11677 (56)	11629–11684 (56)							TAC	TAC	TAC
*nad*6	11680–12113(434)	11678–12111 (434)	11685–12118(434)	TTG/TA	TTG/TA	TTG/TA	145	145	145			
*nad*4L	12114–12345(232)	12112–12343 (232)	12119–12350(232)	ATT/T	ATT/T	ATT/T	78	78	78			
tRNA-Trp	12346–12403 (58)	12344–12401 (58)	12351–12406 (56)							TCA	TCA	TCA
tRNA-Glu	12403–12462 (60)	12401–12458 (58)	12406–12462 (57)							TTC	TTC	TTC
*rrn*S	12463–13159(697)	12459–13154 (696)	12463–13158(696)									
tRNA-SerUCN	13170–13222 (53)	13155–13206 (52)	13166–13218 (53)							TGA	TGA	TGA
AT region	13223–14207(985)	13207–13917 (711)	13218–14153(936)									
tRNA-Asn	14208–14263 (56)	13918–13974 (57)	14154–14210 (57)							GTT	GTT	GTT
tRNA-Tyr	14264–14322 (59)	13975–14029 (55)	14211–14266 (56)							GTA	GTA	GTA

Tcan: *Toxocara canis*, Tcat: *Toxocara cati*, Tmal: *Toxocara malaysiensis*

The mt genome arrangement of *T. canis*, *T. cati *and *T. malaysiensis *are the same as those of *A. suum *and *A. simplex *and almost identical to the genome structures of strongylida nematodes *Ancylostoma duodenale*, *Necator americanus*, *Cooperia oncophora *and rhabditid nematode *Caenorhabditis elegans*, with the exception of the relative position of an AT-rich region and the number of non-coding regions (NCR). In the mt genomes of the three *Toxocara *species, the AT-rich region is located between *trn*S2 and *trn*N, whereas it is positioned between *trn*A and *trn*P in *A. duodenale, N. americanus, Co. oncophora *and *C. elegans*. The only NCR found in *Toxocara *spp. was between *nad*4 and *cox*1, as has been identified in the aforementioned species. The second NCR located between *nad*3 and *nad*5 in the hookworms *A. duodenale *and *N. americanus *was not found in *Toxocara *spp. [9]. The genome structures of these *Toxocara *species differ significantly from those of *Onchocerca volvulus*, *Strongyloides stercoralis*, *Dirofiliria immitis*, *X. americanus *and *T. spiralis *in the location of the AT-rich region, and some tRNA and protein-coding genes. Comparison of the gene arrangements of these three *Toxocara *species with those of the other nine representatives of the secernentean nematodes suggests that *T. canis*, *T. cati *and *T. malaysiensis *are more closely related to *A. suum *and *A. simplex *than to *C. elegans*, *A. duodenale*, *N. americanus*, *Co. oncophora*, *O. volvulus*,*D. immitis *and *S. stercoralis*.

The nucleotide compositions of the entire mtDNA sequences for *T. canis*, *T. cati *and *T. malaysiensis *are biased toward A and T, with T being the most favored nucleotide and C the least favored, in accordance with mt genomes of other nematodes. The content of A+T is 68.57% for *T. canis *(21.9% A, 46.7% T, 22.0% G and 9.4% C), which is the lowest of the nematodes studied to date (Table 2). The content of A+T is 69.95% for *T. cati *(22.3% A, 47.7% T, 20.9% G and 9.1% C) and 68.86% for *T. malaysiensis *(21.7% A, 47.2% T, 22.0% G and 9.1% C), respectively (Table 2).

**Table 2:** Comparison of A+T content of the AT region, protein-coding and rRNA genes of mitochondrial genomes of *Toxocara *species with that of representatives of secernentean nematodes studied to date

**Genes**	**Tcan**	**Tcat**	**Tmal**	**Asu**	**Asi**	**Ad**	**Ov**	**Ss**
*atp*6	68.7	70.2	69.4	71.6	70.7	77.8	73.0	78.4
*cox*1	63.2	64.5	64.4	68.4	66.1	74.0	72.9	72.7
*cox*2	65.0	67.5	65.1	66.7	67.0	69.3	67.0	72.6
*cox*3	65.4	67.3	66.0	68.8	66.3	70.8	69.2	75.5
*cytb*	66.0	68.2	65.3	70.4	67.9	74.3	71.8	75.9
*nad*1	65.4	67.0	66.7	69.5	68.2	74.1	70.0	73.9
*nad*2	70.1	71.1	71.7	73.0	73.3	81.2	74.3	81.3
*nad*3	71.7	73.5	71.7	73.8	75.3	78.3	76.4	78.3
*nad*4	68.0	68.2	67.3	71.0	69.9	78.5	73.2	76.6
*nad*4L	70.8	73.0	73.0	76.5	76.7	80.3	78.6	82.9
*nad*5	69.4	71.2	70.1	72.9	72.1	77.1	72.9	79.6
*nad*6	72.8	74.9	74.9	72.6	71.3	79.3	79.1	82.1
*rrn*S	66.9	68.8	66.4	71.9	71.2	76.6	78.9	69.5
*rrn*L	72.0	73.5	70.1	76.8	76.1	81.1	77.0	77.5
AT-region	79.4	81.3	78.4	84.7	87.2	90.1	85.3	85.0
EmtG	68.57	69.95	68.86	71.95	71.16	76.69	73.30	76.6

Tcan: *Toxocara canis*, Tcat: *Toxocara cati*, Tmal: *Toxocara malaysiensis*, Asu: *Ascaris suum*, Asi: *Anisakis simplex*, Ad: *Ancylostoma duodenale*, Ov: *Onchocerca volvulus*, Ss: *Strongyloides stercoralis*, EmtG: entire mitochondrial genome.

### Protein-coding genes and codon usage patterns

The boundaries between protein-coding genes of mt genomes of *T. canis*, *T. cati *and *T. malaysiensis *were determined by aligning their sequences and by identifying translation initiation and termination codons with those of *A. suum*. For each of the three *Toxocara *species, the lengths of protein-coding genes *cox*3, *nad*1, *nad*2, *nad*3 and *nad*4 are the same as those of *A. suum*, whereas the lengths of genes *atp*6, *nad*4L,*nad*5 and *nad*6 are reduced, and the lengths of *cox*2 and *cyt*b are increased (Table 1) when compared to those of *A. suum*. The length of *cox*1 in *T. canis *and *T. cati *is the same as that of *A. suum*, but the length of *cox*1 in *T. malaysiensis *is increased (Table 3).

**Table 3:** Comparison of protein-coding genes in size, with identity of nucleotides and predicted amino acids for five ascaridoid species

**Gene**	**Sizes of protein-coding genes**					**Identity of nucleotides/amino acids**									
	**Tcan**	**Tcat**	**Tmal**	**Asu**	**Asi**	**Tcan/Tcat**	**Tcan/Tmal**	**Tcan/Asu**	**Tcan/Asi**	**Tcat/Tmal**	**Tcat/Asu**	**Tcat/Asi**	**Tmal/Asu**	**Tmal/Asi**	**Asu/Asi**

*atp*6	598	598	598	600	600	88.8/91.0	85.5/89.9	78.1/79.9	79.1/80.9	89.1/93.0	79.8/79.4	79.4/80.9	80.8/81.4	78.6/82.4	77.7/85.4
*cyt*b	1107	1107	1107	1098	1099	82.2/87.2	83.2/88.9	77.2/76.6	71.3/72.0	83.0/88.6	75.8/75.8	72.1/72.0	74.8/77.4	72.7/73.9	73.7/75.8
*cox*1	1578	1578	1581	1578	1576	89.7/94.1	89.3/93.9	86.1/92.2	83.3/90.7	90.0/94.7	86.4/91.4	83.7/90.5	86.4/92.4	83.1/91.6	83.3/92.6
*cox*2	714	711	711	699	699	87.8/94.9	89.5/95.8	85.1/90.9	80.7/88.4	87.8/95.8	84.7/91.4	80.7/88.8	85.7/90.9	82.1/87.9	82.1/90.1
*cox*3	768	768	768	768	766	86.3/93.7	85.8/92.9	81.0/88.6	79.2/87.5	87.0/94.5	81.8/85.0	80.2/87.1	81.9/87.8	79.0/87.1	79.2/89.0
*nad*1	873	873	873	873	873	85.3/91.7	87.5/93.1	80.2/80.7	81.3/85.9	87.1/94.5	80.8/83.1	82.1/87.2	81.0/81.7	82.0/86.2	80.6/83.4
*nad*2	844	844	844	844	846	84.5/82.9	84.7/85.4	75.9/76.9	72.2/75.8	86.4/88.6	77.6/75.4	72.3/76.9	76.4/75.8	72.4/76.9	75.8/80.1
*nad*3	336	336	336	336	336	87.5/88.3	86.6/85.6	80.4/81.1	82.4/85.6	84.5/84.7	85.1/84.7	81.0/84.7	79.5/79.3	79.5/79.3	78.9/83.8
*nad*4	1230	1230	1230	1230	1230	83.1/87.8	81.4/86.6	75.0/78.2	71.8/76.0	82.0/86.6	74.7/77.8	73.8/78.5	75.0/78.7	72.3/77.5	74.8/80.9
*nad*4L	232	232	232	234	232	85.0/90.9	85.8/85.7	78.1/85.7	76.7/80.5	85.4/89.6	80.7/81.8	79.7/81.8	79.4/79.2	78.0/80.5	80.2/87.0
*nad*5	1582	1582	1582	1585	1582	83.3/87.3	84.3/86.5	76.7/77.6	75.3/77.0	85.8/88.2	76.7/79.1	75.8/79.3	77.7/78.7	77.4/78.1	76.2/82.0
*nad*6	434	434	434	435	435	83.7/85.4	83.0/80.6	72.6/72.2	71.0/72.9	86.4/88.9	76.3/73.6	72.6/73.6	76.1/73.6	72.6/72.2	74.0/80.6
*rrn*S	697	696	696	701	699	89.9	90.4	82.8	81.9	92.2	82.3	84.2	81.5	82.3	83.4
*rrn*L	958	955	955	960	957	83.6	82.4	72.9	69.5	85.2	73.0	72.1	72.5	71.6	71.5
EmtG	14322	14029	14226	14284	13916	85.3	85.7	80.2	78.5	87.1	81.1	80.2	80.5	79.5	80.2

Tcan: *Toxocara canis*, Tcat: *Toxocara cati*, Tmal: *Toxocara malaysiensis*, Asu: *Ascaris suum*, Asi: *Anisakis simplex*, EmtG: entire mitochondrial genome.

The inferred nucleotide and amino acid sequences for each of the 12 mt proteins of *T. canis*, *T. cati *and *T. malaysiensis *were compared with those of *A. suum *and *A. simplex*. The identity of the nucleotide and amino acid sequences is 71~90% and 72.2~95.8%, respectively (Table 3). Based on identity, *cox*1 is the most conserved protein-coding gene, while the *cyt*b is the least conserved. For all 12 proteins, the amino acid sequence identities are higher when compared between each of the three *Toxocara *species than between each of *Toxocara *specis and *A. suum *or *A. simplex*. The identities of the nucleotide and amino acid sequences among five ascaridoid species are higher than those among *C. elegans*, *A. duodenale*, *N. americanus *and *Co. oncophora *(data not shown). These findings reinforce the conclusion that the three *Toxocara *species are genetically more closely related to *A. suum *and *A. simplex *than to *C. elegans*, *A. duodenale*, *N. americanus *and *Co. oncophora*.

The predicted initiation and termination codons for the protein-coding genes of the three *Toxocara *species were compared with those of ascaridoid species (*A. suum *and *A. simplex*) and with selected species representing different nematode orders including the human hookworm *A. duodenale*, filarioid worm *O. volvulus*, and rhabditid species *S. stercoralis*. The most common start codon for three *Toxocara *species is TTG (four of 12 protein-coding genes), followed by ATT (three of 12 protein-coding genes for *T. canis *and *T. malaysiensis*, two for *T. cati*), ATA (two of 12 protein-coding genes for *T. canis *and *T. cati*, one for *T. malaysiensis*), and ATG, GTG, GTT and GTA are used as initiation codons. GTG, which is used in the *cyt*b of *T. canis *and *T. malaysiensis*, and *cox*3 of *T. malaysiensis*, is not used as a start codon in the other nematodes compared. GTA used in the *cox*2 of *T. malaysiensis *is also used as a start codon for *nad*4L in *D. immitis *(data not shown). Seven of the 12 protein-coding genes were predicted to have a TAG or TAA translation termination codon. The remaining protein-coding genes were inferred to end with an abbreviated stop codon, such as TA or T. For the three *Toxocara *species, the 3'-end of most of these genes is immediately adjacent to a downstream tRNA gene (Table 1), which is consistent with the arrangement in the mt genomes of *A. suum *and *A. simplex *[6,15], but in contrast to that of *C. elegans *where both the *nad*1 and *nad*3 genes terminate in T or TA, and are followed by the putative ATT translation initiation codon of their downstream protein-coding genes. The protein-coding gene *nad*6 ended with an abbreviated stop codon TA is followed by the putative ATT translation initiation codon of its downstream protein coding gene *nad*4L, which is similar to *C. elegans*.

In general, the nucleotides of metazoan mt genomes are not randomly distributed, and such nucleotide bias is often associated with unequal usage of synonymous codons. The mt genome nucleotide composition of nematodes is biased toward A and T. The A+T content of protein-coding genes ranged from 63.2% to 74.9% for all three *Toxocara *species (Table 2). This bias in nucleotide composition toward AT (Table 2) affects both the codon usage pattern and amino acid composition of proteins. In these three species examined, all 64 possible codons were used, and the most frequently used codon was TTT (Phe) while the least used codon was CGC (Arg). The preferred nucleotide usage at the third codon position of *Toxocara *mt protein-coding genes reflects the overall nucleotide composition of the mtDNA. At this position, T is the most frequently used, and C the least frequently used. The codons ending in G have higher frequencies than the codons ending in A, which is similar to *A. suum*, but opposite to *C. elegans*, *A. duodenale *and *N. americanus*.

The protein-coding genes of the three *Toxocara *genomes are biased toward using amino acids encoded by T-, A- and G-rich codons (data not shown). The AT-rich codons represent amino acids Phe, Ile, Met, Tyr, Asn or Lys, and the GC-rich codons represent Pro, Ala, Arg or Gly. T-rich codons (with ≥2 Ts in a triplet) comprise Phe (13.4% TTT and 0.6% TTC), Leu (9.0% TTG, 3.8% TTA, and 1.5% CTT), Ile (5.3% ATT), Val (6.2% GTT), Tyr (4.1% TAT), Ser (4.0% TCT), and Cys (1.5% TGT), and account for approximately half (49.6%) of the total amino acid composition. A- and G-rich codons (with ≥2 As and Gs, respectively) represent 9.7% and 13.3% of the total amino acid composition, respectively (data not shown). In contrast, the proportion of C-rich codons (with ≥2 Cs) is much lower (4.0%). This codon bias against C is even more evident when only the third codon positions are considered in both four fold and two fold degenerate codon families. When the frequencies of synonymous codons within the AT-rich group were compared, the frequency was always decreased if the third position was substituted with a C. For instance, the relative frequencies of codons for Phe are 13.4% TTT and 0.6% TTC, respectively. This result suggests that the third codon positions mostly reflect mutational bias against C. A greater translational efficiency has also been considered to be a potential cause underlying observed codon usage bias [27].

### Transfer RNA genes

Twenty-two tRNA sequences (ranging from 52 to 63 bases in size; see Table 1) were identified in the mt genomes of the three *Toxocara *species. Their putative secondary structures (not shown) are similar to those of other nematode mtDNAs [6,7,9-11,15], with the exception of *T. spiralis *[8], and differ from the conventional cloverleaf-like structures found in other metazoan mtDNA molecules. Common features of the predicted secondary structures (not shown) of 22 tRNA genes in *T. canis*, *T. cati *and *T. malaysiensis *mtDNA include a 7 bp amino-acyl stem, a 4 bp DHU stem with a 4–8 bp loop, a 5 bp anticodon stem with a loop of 7 bp (a T always preceding an anticodon as well as an A or a G always following an anticodon), and a TV replacement loop of 6–12 bp with some exceptions, in accordance with other nematodes. The exception is *trn*S1 (AGN) in which the DHU-arm is lacking.

### Ribosomal RNA genes

The *rrn*S and *rrn*L genes of the three roundworm species were identified by sequence comparison with those of *A. suum*. The *rrn*S is located between *trn*E and *trn*S (UCN), and *rrn*L is located between *trn*H and *nad*3. The two genes are separated from one another by the protein-coding genes *nad*3, *nad*5, *nad*6 and *nad*4L. The size of the *rrn*S gene is 697 bp for *T. canis*, 696 bp for *T. cati *and 696 bp for *T. malaysiensis*. The size of the *rrn*L gene is 958 bp for *T. canis*, 955 bp for *T. cati *and 955 bp for *T. malaysiensis*. The sizes of the two rRNA genes for the three *Toxocara *species are similar to those of other nematodes (Table 1). The A+T contents of the *rrn*S for *T. canis*, *T. cati *and *T. malaysiensis *are 66.9%, 68.8% and 66.4%, respectively, whereas those of the *rrn*L are higher (72.0%, 73.5% and 70.1%, respectively), and A+T contents of the two genes are the lowest among the nematodes studied to date (Table 2). Sequence identity in the *rrn*S and *rrn*L genes is 87.5% and 83.6% between *T. canis *and *T. cati*, 87.9% and 82.4% between *T. canis *and *T. malaysiensis*, and 91.4% and 85.2% between *T. cati *and *T. malaysiensis*, respectively.

### Non-coding regions

Like *A. suum *and *A. simplex*, the longest non-coding region (AT-region) in the three *Toxocara *mt genomes is located between the *trn*S2 and *trn*N. Their sizes are 985 bp (*T. canis*), 711 bp (*T. cati*) and 936 bp (*T. malaysiensis*), and A+T contents are 79.4% (*T. canis*), 81.3% (*T. cati*) and 78.4% (*T. malaysiensis*), respectively, which are significantly lower than the comparable NCRs of nematodes studied to date (Table 2). Repeated sequence motifs (CR1-CR6) present in the *C. elegans *AT-rich region [6] are not found in *Toxocara *spp. However, there are a lot of AT dinucleotide repeats in the AT-region of *Toxocara *mt genomes of which the longest consists of repeat units (34 base pairs). Similar AT dinucleotide repeats has been found in *A. suum *[6]. The function or role of these AT repeats is currently unknown [6,7]. Although nothing is yet known about the replication process(es) in the mtDNA of parasitic nematodes, the high A+T content and the predicted structure of the AT-rich non-coding region suggests an involvement in the initiation of replication [28].

For the three roundworm species, the second longest non-coding region is located between genes *cox*1 and *nad*4, as in the mt genomes of *A. suum *[6]. Its length is 111 bp (*T. canis*), 116 bp (*T. cati*) and 112 bp (*T. malaysiensis*), with an A+T content of 86.2%, 74.1% and 74.1%, respectively, and is shorter than that of *A. suum *(117 bp). The non-coding region for the three *Toxocara *species could form a hairpin loop structure (AATTTTTAAAAATT).

### Phylogenetic analyses

**Figure 1:**
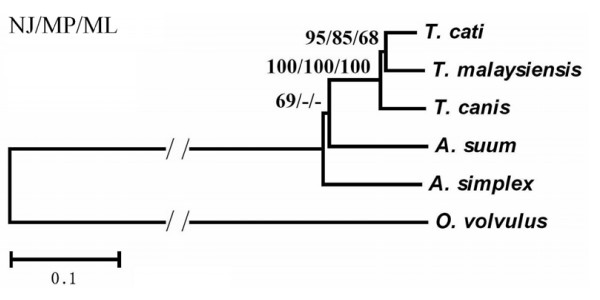
Inferred phylogenetic relationship among the five ascaridoid species (*Toxocara canis, T. cati, T. malaysiensis*, *Ascaris suum*, *Anisakis simplex*) derived from neighbour joining (NJ), maximum parsimony (MP) and maximum likelihood (ML) analyses of amino acid sequences of 12 protein-coding genes from their mitochondrial genomes, using one filarioid species (*Onchocerca volvulus*) as outgroup. The numbers along branches indicate bootstrap values resulting from different analyses in the order: NJ/MP/ML. Values lower than 50 are given as "-".

The final alignment of the amino acid sequences of 12 proteins for the six taxa (*T. canis, T. cati, T. malaysiensis*, *A. suum*,*A. simplex *and *O. volvulus*) yield 3516 characters (2079 variable, 339 parsimony-informative). In all three phylogenetic analyses, three *Toxocara *species were clustered together (Fig. 1). *T. malaysiensis*, the recently described *Toxocara *species from cat [26], was inferred to be the sister species of *T. cati *with high bootstrap values. This result was consistent with that of a previous study [25] which used sequences of internal transcribed spacers of nuclear ribosomal DNA, thus providing mt DNA evidence for the validity of *T. malaysiensis *as an ascaridoid of cats. *T. malaysiensis *is more closely related to *T. cati*, the common ascaridoid of cats, than to *T. canis*, the common ascaridoid of canids.

*Toxocara *species was resolved being more closely related to *A. suum *than to *A. simplex *with moderate support in the phylogenetic analyses, which was consistent with results of previous morphological and molecular studies [29,30]. But relationship between *A. suum *and *A. simplex *was poorly inferred in the MP and ML analyses (Fig. 1).

## Conclusion

*Toxocara *species are the important socio-economic parasites because they have significant impact on human health. The determined mt genomes of the three roundworms, *T. canis*, *T. cati *and *T. malaysiensis*, add the mtDNA data to the order Ascaridida, which includes a broad range of parasites of major socio-economic importance. Determination of the complete mt genome sequences for three *Toxocara *species of human and animal health significance provides a foundation for studying the systematics, population genetics and ecology of these and other nematodes of socio-economic importance.

## Methods

### Parasites and DNA extraction

The ascaridoid nematodes used in the present study were from Zhanjiang city for *T. canis*, Changsha city for *T. cati *and Guangzhou city for *T. malaysiensis*, China, respectively. Adult nematodes of three *Toxocara *species were obtained from the intestines of dogs and/or cats, washed in physiological saline, identified primarily based on morphological characters to species, fixed in 70% (v/v) ethanol and stored at -20°C until use. Total DNA was isolated from individual nematodes using sodium dodecyl-sulphate/proteinase K treatment, followed by spin-column purification (Wizard Clean-Up, Promega). The specific identity of each individual nematode was verified by species-specific PCR amplification using the sequences of the first and/or second internal transcribed spacers (ITS-1 and/or ITS-2) of ribosomal DNA (rDNA) as the species-specific genetic markers [31,32].

### Long-PCR amplification and sequencing

Using primer set 5F/40R (~9 kb region) and 39F/42R (~6 kb region) [33], the entire mt genome of each *Toxocara *species was amplified in two overlapping fragments by long-PCR from approximately 20 ng of total genomic DNA purified from an individual nematode, respectively. The primers were designed to mt sequences found to be relatively conserved among *A. suum *and *C. elegans*. Primer 5F (forward, 5'-TATGAGCGTCATTTATTGGG-3') and its complementary primer 42R (reverse, 5'-CCCAATAAATGACGCTCATA-3') were designed to the *nad*1 gene, while primers 39F (forward, 5'-TAAATGGCAGTCATTAGCGTGA-3') and 40R (reverse, 5'-GAATTAAACTAATATCACGT-3') were designed to the *rrn*L gene [33]. Long-PCR cycling conditions used were 92°C for 2 min (initial denaturation), then 92°C for 10 s (denaturation), 50°C for 30 s (annealing), and 60°C (for 6 kb) or 68°C (for 9 kb) for 10 min (extension) for 10 cycles, followed by 92°C for 10 s, 50°C for 30 s, and 60°C (for 6 kb) or 68°C (for 9 kb) for 10 min for 20 cycles, with a cycle elongation of 10 s for each cycle and a final extension at 60 or 68°C for 7 min. Each PCR reaction yielded a single band detected in a 0.8% (w/v) agarose gel upon ethidium-bromide staining (not shown). PCR products were sent to TaKaRa Company (Dalian, China) for sequencing using a primer walking strategy, and the following primers were used for sequencing the complete genomes of three *Toxocara *species: MW1F (5'-ATATTAGATTGTAAATCTAAAG-3'), MW5-1R (5'-ACACGAAAAACCCAAAAATT-3'), MW5-1F (5'-ATTATGTCGTCTTGATAAGGC-3'), MWTCT62F (5'-GCTATTATGATGGAGTGTTTTGTG-3'), MW62-1F (5'-GTTACCTTTTTTACCTGTGCTTGT-3'), MW40R (5'-CTGGTGAACAGATATCAAAGGACATCATC-3'), MW5-2F (5'-TAGATTGGTATATTTTGATTTTAACTTGTCT-3'), MW79F (5'-GGGTTTTAATTTATTTCGTTT-3'), MW78R (5'-GTAGTAGGTGCTTCTACATG-3'), MW62-3F (5'-TCTACTGAGTCTTTTAATTGAG-3'), MWXIAYOU (5'-CCCAATAAATGACGCTCATA-3'), MWTM-1 (5'-GTATATTGCTAGCGGGTCTTTT-3'), MW0209-115 (5'-AAACTGGTGAACAGATATCAAA-3'), MW4F (5'-GGAGTAAGTTGTAGTAAAGTAGA-3'), MW38R (5'-AGAAAAAGCAATCTCATAAGAA-3'), MW1021-3 (5'-TAAATGGCAGTCTTAGCGTGA-3'), MW5-3F (5'-TTAGATTGTAAATCTAAAGA-3'), MWND4-2F (5'-TTACTATCTGATTTTTTAT-3'), MWND4-1F (5'-TTATTAGTTTGGTTTATTTT-3'), MW6K2F (5'-TTAGGAGAAATTTCCGTAGTTT-3'), MW9K1F (5'-ATCAATTCGGCTTATTATTTAA-3'), MW62R (5'-ATAGCATAGACCATGCCCAAAG-3'), MW62-2F (5'-ACTTTTCAGAAGTTACCTTTTTT-3'), MW51F (5'-TGGTTTTCTTACTTTATTTGTTT-3'), MWND1R (5'-TATCATAACGAAAACGAGG-3'), MW1R (5'-CCAGGAACCAAAATAAAAGA-3'), MWTM62F (5'-TTTCTTACTTTTCAGAAGTT-3'), MWSRNAF1 (5'-TAATCGGCTAGACTTTATAAACT-3'), MWND1-1R (5'-ACAAACCACTCCAAATACAT-3'), MWSRNAR (5'-TAATGAGGGCTCTCAATTACT-3'), and MWSRNAF2 (5'-TTAAAAGAGCAGGAGTAAAGTT-3').

### Sequence analyses

Sequences were assembled manually and aligned against the complete mt genome sequence of *A. suum *(GenBank™ accession number NC001327) using the program Clustal X to identify gene boundaries. The open-reading frames and codon usage profiles of protein-coding genes were analyzed using the program MacVector 4.1.4 (Kodak, version 4.0). Translation initiation and translation termination codons were identified based on comparison with those reported previously for *A. suum*. The amino acid sequences inferred for the mt genes of three ascaridoids were aligned with those of *A. simplex *(GenBank™ accession number AY994157) and *A. suum *using Clustal X. Based on pairwise alignments, amino acid identity (%) was calculated for homologous genes. Codon usage was examined using the relationships between the nucleotide composition of codon families and amino acid occurrence, where the genetic codons are partitioned into AT-rich codons (i.e. those which are AT-rich at the first two codon positions), GC-rich codons (those which are GC-rich at the first two codon positions) and unbiased codons. For analyzing ribosomal RNA genes, putative secondary structures of 22 tRNA genes were identified using tRNAscan-SE [34], or by recognizing potential secondary structures and anticodon sequences by eye by aligning sequences with those of *A. simplex *and *A. suum*.

### Phylogenetic analyses

Phylogenetic analyses were performed using the five ascaridoid species (*T. canis, T. cati, T. malaysiensis*, *A. suum*,*A. simplex*) as ingroups, and one filarioid species (*O. volvulus*) serving as outgroup (GenBank™ accession number AF015193), based on amino acid sequences of 12 protein-coding genes. Amino acid sequences for each gene were individually aligned using Clustal X under default setting, and then concatenated into single alignments for phylogenetic analyses. Standard unweighted maximum parsimony (MP) were performed in PAUP* 4.0b10 [35] using heuristic searches with tree-bisection-reconnection branch swapping and 1000 random-addition sequence replicates with 10 trees held at each step. The Dayhoff matrix model was utilized in the analyses of neighbour joining (NJ), implemented by MEGA 3.1 [36], and maximum likelihood (ML) implemented by PhyML 2.1 [37]. Branch supports were estimated by bootstrap analysis of 1000 replicates for NJ and MP trees, and 100 replicates for ML tree.

## Authors' contributions

MWL performed the majority of the study and analyzed the data, and contributed to drafting of the manuscript. RQL and HQS performed part of the study, and provided technical assistance. XYW contributed to the analysis of the data and helped in revising the manuscript. XQZ conceived and designed the research plan, participated in all aspectes of the study, provided funds, supervised the research, and took the lead on drafting the manuscript. All authors read and approved the final manuscript.
